# RP11-439C15.4 inhibits the malignant progression of hepatocellular carcinoma via binding to DHX9 and facilitating its degradation: RP11-439C15.4 inhibits HCC progression by downregulating DHX9

**DOI:** 10.3724/abbs.2025122

**Published:** 2025-07-17

**Authors:** Xuejiao Li, Zhongying Hu, Yina Sun, Tingting Wang, Xijing Yan, Qiang You, Kunhua Hu, Jia Yao, Xiaofeng Yuan, Rong Li

**Affiliations:** 1 Guangdong Provincial Key Laboratory of Liver Disease Research the Third Affiliated Hospital of Sun Yat-sen University Guangzhou 510630 China; 2 Department of Hepatic Surgery and Liver Transplantation Center of the Third Affiliated Hospital of Sun Yat-sen University; Organ Transplantation Research Center of Guangdong Province Guangdong Province Engineering Laboratory for Transplantation Medicine. Guangzhou 510630 China; 3 Department of Laboratory Medicine the Third Affiliated Hospital of Sun Yat-sen University Guangzhou 510630 China; 4 Department of General Intensive Care Unit Lingnan Hospital the Third Affiliated Hospital of Sun Yat-sen University Guangzhou 510530 China; 5 Department of Breast and Thyroid Surgery Lingnan Hospital the Third Affiliated Hospital of Sun Yat-Sen University Guangzhou 510530 China

**Keywords:** hepatocellular carcinoma, RP11-439C15.4, DHX9, malignant progression

## Abstract

Long noncoding RNAs (lncRNAs) play crucial roles in the occurrence and progression of hepatocellular carcinoma (HCC), but the functions and molecular mechanisms of large lncRNAs remain unclear. In this study, HCC data from The Cancer Genome Atlas (TCGA) and 116 HCC cases from our clinical center are used to identify a novel lncRNA, RP11-439C15.4, which is significantly downregulated in HCC. This downregulation is associated with poor prognosis in HCC patients. A series of *in vitro* and *in vivo* experiments demonstrate that RP11-439C15.4 significantly inhibits the proliferation, invasion, migration and sorafenib resistance of HCC cells. Further mechanistic investigations reveal that RP11-439C15.4 interacts with DExH-Box Helicase 9 (DHX9) to increase its ubiquitination and accelerate the degradation of DHX9, ultimately suppressing HCC progression. Modulation of DHX9 significantly reverses the effects of RP11-439C15.4 in HCC. In conclusion, this study identifies RP11-439C15.4 as a tumor suppressor and elucidates the regulatory mechanism of the RP11-439C15.4/DHX9 axis in HCC, providing valuable insights into the mechanisms of HCC progression and potential therapeutic targets.

## Introduction

Hepatocellular carcinoma (HCC), the main type of primary liver cancer, is characterized by high morbidity, high mortality, and a poor prognosis, making it a leading cause of cancer-related deaths worldwide [1]. Projections suggest that the incidence of HCC will surpass one million cases annually after 2025, with an estimated one million deaths attributed to the disease each year by 2030 [2]. The insidious onset of HCC and the absence of effective early diagnostic methods have resulted in many patients being diagnosed at advanced stages with metastasis, contributing to the poor prognosis and a five-year survival rate of only 18% [ 3, 4] . Thus, there is an urgent need for improved early detection, treatment, and research into the biological mechanisms underlying HCC.

Long non-coding RNA (lncRNA) is a class of non-coding RNA molecules exceeding 200 nucleotides in length that lack protein-coding capacity. Previous studies have demonstrated the importance of lncRNAs in diverse biological processes, including epigenetic regulation, cell cycle regulation, and cell differentiation regulation, establishing them as prominent areas of genetics research. Similarly, lncRNAs have been implicated in the development and progression of various cancers, including HCC. Research has indicated that lncRNAs can modulate HCC cell proliferation, migration, and invasion through interactions with DNA, proteins, or other RNAs, suggesting their potential as biomarkers for tumor diagnosis and treatment [5]. However, the lncRNAs currently identified in HCC represent only a fraction of the total, and the functions of many remain to be elucidated. A comprehensive investigation of lncRNA functions and their underlying molecular mechanism in HCC is essential for a deeper understanding of HCC pathogenesis and the identification of novel diagnostic and therapeutic targets.

In this study, via the use of RNA sequencing data from HCC samples in TCGA and HCC clinical samples from our center, we identified for the first time that the lncRNA RP11-439C15.4 is significantly downregulated in HCC and is closely associated with patient prognosis. *In vitro* and *in vivo* functional experiments demonstrated the ability of RP11-439C15.4 to inhibit HCC cell growth, migration, and invasion and to increase HCC cell sensitivity to sorafenib-induced cell death. Mechanistically, RP11-439C15.4 can interact with DExH-box helicase 9 (DHX9), a member of the DEAH-containing family of RNA helicases, which has been reported to play important roles in HCC progression and drug resistance. The interaction of RP11-439C15.4 with DHX9 significantly upregulated DHX9 ubiquitination, increasing its degradation via the ubiquitin‒proteasome pathway and thereby inhibiting the malignant progression of HCC.

## Materials and Methods

### Tissue sample

A total of 116 HCC patients provided written informed consent at the Third Affiliated Hospital, Sun Yat-Sen University (Guangzhou, China), from 2017–2019. This study was approved by the Institutional Research Ethics Committee (RG2023-151-01). Informed written consent was obtained from all participants as well. None of the enrolled patients had received preoperative chemotherapy or radiotherapy. All the tumor tissues were confirmed to be HCC by hematoxylin and eosin (H&E) staining. Normal liver tissues were collected at a standard distance (2 cm) from the HCC resection margin and confirmed by pathological evaluation.

### Cell culture

The human HCC cell lines (97H, Hep3B, SK-Hep1, Huh-7, HCC-LM3, PLC-PRF-5, and HepG2) were obtained from Cellcook Biotech Co., Ltd. (Guangzhou, China). These cell lines were cultured in Dulbecco’s modified Eagle’s medium (DMEM) supplemented with 10% fetal bovine serum (FBS) and 1% penicillin-streptomycin to prevent microbial contamination. The normal liver cell line THLE-2 was purchased from Guang Zhou JENNIO Biotech Co., Ltd. (Guangzhou, China) and maintained in BEGM (BEGM Bullet Kit; Lonza/Clonetics Corporation, Walkersville, USA). All the cell lines were cultured in a 5% CO _2_ incubator at 37°C and routinely tested for mycoplasma contamination every 6 months. The identity of the cell lines was confirmed via short tandem repeat (STR) profiling.

### RNA extraction and real-time quantitative PCR (qRT-PCR)

Total RNA was extracted via TRIzol reagent (Invitrogen, Carlsbad, USA), and its concentration and purity were assessed via a NanoDrop 2000 spectrophotometer (Thermo Fisher Scientific, Waltham, USA). Reverse transcription was performed via GoScript ^TM^ Reverse Transcription System (Promega, Madison, USA) to generate cDNA. Quantitative reverse transcription PCR (qRT-PCR) was performed via 2× SYBR Green qPCR Master Mix (AG11701; AG, Changsha, China) on an LC480 real-time PCR system (Roche, Basel, Switzerland). Glyceraldehyde-3-phosphate dehydrogenase ( *GAPDH*) was used as an internal control. All the experiments were performed in triplicate. The primer sequences are listed in Supplementary Table S1.

### Western blot

Western blot was performed according to previously described methods [ 6, 7] . Briefly, total protein was extracted via RIPA lysis buffer (Beyotime, Shanghai, China). The protein concentration in the cell lysates was quantified via a BCA protein assay kit (Thermo Fisher Scientific). Equal amounts of protein were separated by 10% SDS-PAGE and subsequently transferred onto PVDF membranes. The membranes were blocked with 5% skim milk for 1 h at room temperature (RT) and then incubated with the indicated primary antibodies (including antibodies against DHX9 (1:500; Cell Signaling Technology, Danvers, USA) and β-actin (1:2000; Cell Signaling Technology) at 4°C overnight. The membranes were then incubated for 1 h at room temperature with the corresponding HRP-labeled secondary antibodies. Following incubation with an enhanced chemiluminescence (ECL) substrate, the blots were visualized via an enhanced chemiluminescence (ECL) imaging system (Tanon-5200, Shanghai, China). The experiments were performed in triplicate.

### Intracellular RNA localization analysis

Nuclear and cytoplasmic RNAs were isolated from the indicated cells via the PARIS™ Kit (Thermo Fisher Scientific) and subjected to qRT-PCR analysis. *U3* and *GAPDH* served as internal references for the nuclear and cytoplasmic fractions, respectively. The following formula was used to calculate the percentage of RNA in each fraction: Cytoplasm% = 2^CT(nuclear)/(2^CT(cytoplasm) + 2^CT(nuclear)), Nuclear%=1 – Cytoplasm%. The primer sequences are listed in Supplementary Table S1.

### Fluorescence *in situ* hybridization (FISH)

The RP11-439C15.4 probe and related FISH kit were designed and manufactured by RIB-BIO (Guangzhou, China). FISH assays were conducted according to the manufacturer’s instructions. Briefly, cells were cultured overnight in 8-well chamber slide plates and then fixed with 4% paraformaldehyde for 30 min before being treated with 0.5% Triton X-100 for 5 min. After being blocked with prehybridization solution for 30 min, the cells were incubated with the RP11-439C15.4 probe at 37°C overnight. The cell slides were then washed sequentially in 4× SSC, 2× SSC, and 1× SSC for 5 min each and counterstained with DAPI for 10 min. The FISH results of RP11-439C15.4 were observed via a confocal microscope.

### Plasmids and siRNA transfection

The RP11-439C15.4 overexpression plasmid was constructed via the retroviral plasmid vector pLVX-Puro. RP11-439C15.4 silencing was achieved via a corresponding lncRNA smart silencer purchased from RIB-BIO (Guangzhou, China). The pEnCMV-DHX9 (human)-3× FLAG-SV40-neo plasmid was obtained from MiaoLing (Wuhan, China). *DHX9* silencing was performed via a siRNA designed and produced by GenePharma (Suzhou, China). Plasmid and siRNA transfections were carried out via jetPRIME (Polyplus-transfection, Illkirch, France) following the manufacturer’s instructions. The sequences of the RP11-439C15.4 cloning primers, the RP11-439C15.4 lncRNA smart silencer, and the DHX9 siRNA are provided in Supplementary Table S1.

### Cell proliferation assays

Cell proliferation was evaluated via CCK-8, colony formation, and EdU incorporation assays. For the CCK-8 assay, cells were seeded in 96-well plates at a density of 600 cells/well and transfected with the indicated plasmids or siRNAs. At the designated time points, the culture medium was removed, and 10 μL of CCK-8 solution (KeyGEN BioTECH, Nanjing, China) mixed with 90 μL of fresh cell culture medium was added to each well. The cells were incubated for 2 h at 37°C, and the absorbance was measured at 450 nm via a spectrophotometer. For the colony formation assays, cells were seeded in 6-well plates at a density of 800 cells/well and cultured for two weeks at 37°C in a humidified atmosphere containing 5% CO2. Colonies were then washed with PBS, fixed with 4% paraformaldehyde, and stained with 0.1% crystal violet solution for 30 min at room temperature. After washing with deionized water (ddH _2_O) and drying, colonies were quantified via Image-Pro Plus 6.0 (Media Cybernetics, Bethesda, USA). For the EdU incorporation assays, a Fluor488-EdU kit (KeyGEN Bio TECH) was used. EdU staining was performed according to the manufacturer’s protocol, and images were acquired via an inverted fluorescence microscope (Carl Zeiss, Jena, Germany). The percentage of EdU-positive cells was calculated as the ratio of the number of EdU-positive cells to the number of DAPI-positive cells.

### Cell migration and invasion assays

To evaluate cell migration and invasion, wound-healing and transwell assays were performed, respectively. For the wound-healing assay, cells were seeded in 6-well plates (4×10 ^5^ cells/well), and after overnight incubation, linear wounds were created via a 10-μL pipette tip. Images of the wound closure were captured at 0, 12, and 24 h. For the Transwell invasion assay, 24-well Transwell plates with Matrigel-coated upper chambers were used, where the chambers were coated with 50 μL of 10% Matrigel and incubated at 37°C for at least 30 min. A total of 4×10 ^4^ cells were seeded in the upper chamber with serum-free medium, and the lower chamber contained medium supplemented with 10% FBS. After 12–24 h, non-invaded cells were removed from the upper chamber, and invading cells in the lower chamber were fixed with 4% paraformaldehyde, stained with 0.1% crystal violet, washed, and photographed via an inverted fluorescence microscope. Cell quantification was performed via Image-Pro Plus 6.0 software.

### TUNEL assay

Terminal deoxynucleotidyl transferase (TdT) deoxyuridine triphosphate (dUTP) nick end labeling (TUNEL) is an assay typically used to detect and quantify cell death. In this study, TUNEL assays were performed via a One Step TUNEL Apoptosis Kit (green fluorescence) (Beyotime) according to the manufacturer’s protocol. Briefly, cells were seeded in 8-well chamber slide plates at a density of 2.5×10 ^4^/chamber. Following the indicated treatment, the cells were fixed with 4% paraformaldehyde for 30 min and permeabilized with 0.5% Triton X-100 for 5 min at room temperature. The TUNEL detection solution was then prepared and incubated with the cells at 37°C for 1 h in the dark. After counterstaining with DAPI, images were acquired via an inverted fluorescence microscope (Carl Zeiss).

### *In vivo* tumor xenograft model and lung metastasis models

All the mice (6-week-old) used in this study were NCG mice purchased from GemPharmatech (Nanjing, China). The mice were housed under specific pathogen-free (SPF) conditions with a controlled temperature of 25°C, humidity between 40% and 70%, and a 12 h light/dark cycle. To assess the effects of RP11-439C15.4 on tumor proliferation and metastasis *in vivo*, SK-Hep1 cells stably transfected with either RP11-439C15.4 or the negative control were administered to NCG mice ( *n*  = 5 per group) via subcutaneous injection (3 × 10 ^6^ cells) or tail vein injection (1 × 10 ^6^ cells). Tumors were measured via a Vernier caliper once a week. Four to six weeks post-injection, the mice were euthanized. Livers and lungs were harvested for H&E staining. All the animal experiments were conducted in accordance with Chinese legislation concerning the use of experimental animals and were approved by the Institutional Animal Care and Use Committee (IACUC), Jennio Biotech Co., Ltd. (JENNIO-IACUC-2023-A061).

### RNA pull-down and silver staining assays

To identify the proteins that bind to RP11-439C15.4, RNA pull-down assays were performed. The RNA was transcribed *in vitro* via the T7 High Yield RNA Transcription Kit (Vazyme, Nanjing, China) to generate sufficient quantities of sense and antisense RP11-439C15.4 RNA. Subsequently, RNA pull-down assays were conducted via the Pierce Magnetic RNA-Protein Pull-Down Kit (Thermo Fisher Scientific) following the manufacturer′s instructions. The protein products obtained from the RNA pull-down assays were analyzed using SDS-PAGE followed by silver staining via a Pierce Silver Stain Kit (Thermo Fisher Scientific) according to the manufacturer’s protocol. Potential RP11-439C15.4-binding partners were then identified via a UPLC-Q Exactive HF-X mass spectrometer system (Thermo Scientific, Bremen, Germany) following a standard method [8]. Briefly, initial RNA pull-down products were denatured in SDT buffer (4% w/v SDS, 100 mM Tris/HCl, 1 mM DTT, pH 7.6) at 100°C for 2 min. Samples were then transferred to Microcon UFC201024 devices (Millipore), mixed with 200 μL of 8 M urea, and concentrated by centrifugation (14,000 *g*, 20°C, 40 min; consistent parameters applied for all subsequent centrifugation steps). The concentrate was washed twice with 200 μL of 8 M urea. Alkylation was performed by adding iodoacetamide (IAA) to a final concentration of 25 mM, followed by incubation at room temperature for 30 min in the dark. The alkylated sample was washed twice with 100 μL of 8 M urea and twice with 50 mM TEAB. Proteolytic digestion was then carried out overnight (16–18 h) at 37°C using trypsin. The resulting peptides were separated by UHPLC and analyzed by label-free mass spectrometry on a QE HF-X MS. MS/MS spectra were acquired with the following parameters: resolution 15,000; AGC target <2 × 10 ^4^; maximum isolation time 30 ms; normalized collision energy 27%. Each experimental group included three biological replicates. Raw MS files were processed using MaxQuant software (Thermo Fisher Scientific) with Proteome Discoverer for specific protein analysis. Database searches were performed against *C*. *sinensis* proteome with the following parameters: precursor mass tolerance ± 15 ppm; fragment ion tolerance ± 0.5 Da; dynamic modification oxidation (M); maximum two missed cleavages; FDR thresholds of 1% at both PSM and protein levels, enforced using a target-decoy approach. The primers used for *in vitro* transcription of sense and antisense RP11-439C15.4 are listed in Supplementary Table S1.

### Co-immunoprecipitation

Co-immunoprecipitation (Co-IP) assays were performed as previously described [ 9] . Briefly, the indicated cells were lysed in NP-40-containing lysis buffer supplemented with a protease inhibitor cocktail (Merck, Darmstadt, Germany). Pierce anti-Flag magnetic agarose (Merck) was then added to the cell lysate, which was subsequently incubated overnight at 4°C. The beads were subsequently washed three times with IP wash buffer (150 mM NaCl, 10 mM HEPES pH 7.4, and 0.1% NP-40). Finally, proteins were eluted from the beads via RIPA lysis buffer (Beyotime) and subjected to western blotting.

### Cycloheximide (CHX) chase assay

In this study, a CHX chase assay was used to monitor intracellular protein degradation and determine the half-life of DHX9 in SK-Hep1 cells. The indicated cells were seeded at a density of 1.25 × 10 ^5^ cells per well in a 12-well plate and subsequently treated with 10 μM CHX. Whole-cell extracts were collected at the indicated time points (0, 6, 12, and 24 h). DHX9 protein levels in these extracts were then assessed by western blot.

### Immunohistochemical staining

Immunohistochemical (IHC) staining assays were performed as previously described [6]. Briefly, paraffin-embedded tumor tissues (4 μm) were deparaffinized and rehydrated through a graded ethanol series. Antigen retrieval was performed via either sodium citrate buffer or ethylenediaminetetraacetic acid (EDTA) according to the primary antibody protocol. The tissue sections were subsequently blocked with QuickBlock Blocking Buffer (Beyotime) for 30 min at room temperature and then incubated overnight at 4°C with the indicated primary antibodies. Secondary antibody incubation and DAB chromogen development were performed via the Dako REAL EnVision Detection System (Copenhagen, Denmark). Images were acquired via a Tissue FAXS PLUS system (TissueGnostics, Vienna, Austria). The primary antibodies used included anti-DHX9 (1:200; Proteintech Group, Chicago, USA), anti-Ki67 (1:300; Servicebio, Wuhan, China), and anti-CD31 (1:500; Servicebio) antibodies.

### Statistical analysis

Statistical analyses were performed via SPSS version 13.0 (IBM, New York, USA) and GraphPad Prism version 8 (GraphPad Software, La Jolla, USA). Correlations between RP11-439C15.4 expression levels and clinicopathological features were analyzed via the chi-square test. Survival curves were generated via the Kaplan‒Meier method and compared via the log-rank test. Student’s *t* test was used for comparison between two independent groups. The data are presented as the mean±standard deviation (SD) from three independent experiments. A *P* value of less than 0.05 was considered statistically significant.

## Results

### RP11-439C15.4 is downregulated in HCC and is positively correlated with patient prognosis

Recent studies have increasingly demonstrated the important roles of lncRNAs in the initiation and progression of HCC, suggesting their potential as promising targets for diagnosis, treatment, and prognosis assessment. Currently, the lncRNAs with well-defined functions and molecular mechanisms in HCC represent only a small fraction of the total. Further investigation is needed to elucidate the functions of the numerous dysregulated lncRNAs observed in HCC.

**Figure 1:**
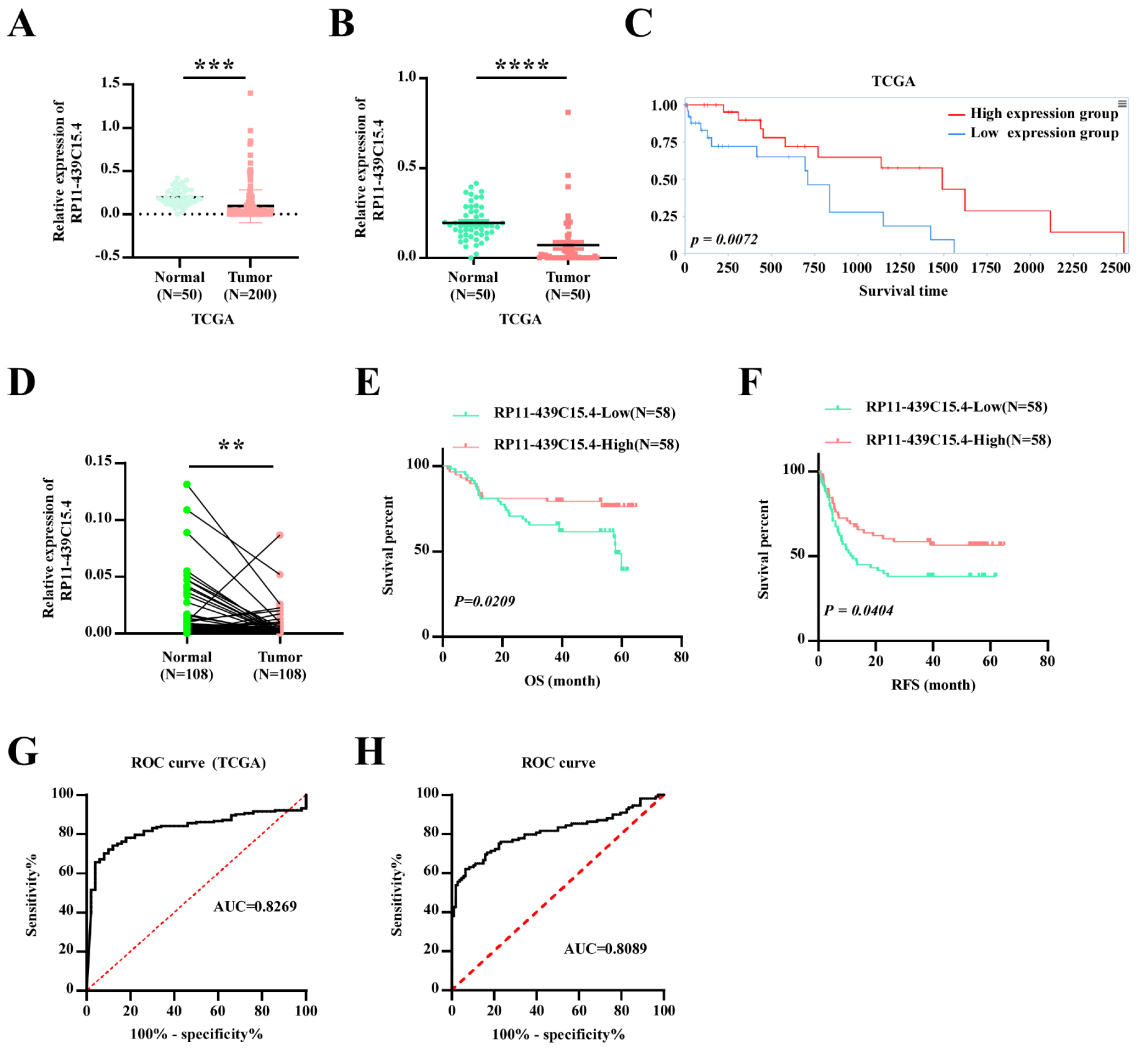
Figure 1 The expression and prognostic significance of RP11-439C15.4 in HCC (A) Analysis of RP11-439C15.4 expression in normal liver tissues and all HCC tissues in the TCGA database. (B) Analysis of RP11-439C15.4 expression in HCC tissues and paired non-tumor tissues in the TCGA database. (C) Kaplan-Meier analysis of OS between the low and high RP11-439C15.4 expression groups of HCC patients collected from the TCGA. The grouping of patients was based on the median RP11-439C15.4 RNA level. (D) Analysis of RP11-439C15.4 expression in 108 pairs of HCC tissues and paired non-tumor tissues from our clinical center. (E) OS curves of 116 HCC patients with low or high RP11-439C15.4 expression in our clinical center. (F) RFS curves of 116 HCC patients with low or high RP11-439C15.4 expression in our clinical center. (G,H) ROC curve of RP11-439C15.4 in HCC generated with the TCGA dataset (G) and our clinical center data (H). An area under the curve (AUC) > 0.7 was considered to indicate diagnostic value. P < 0.05 was considered statistically significant. **P < 0.01, ***P < 0.001 and ****P < 0.0001.

This study utilized HCC datasets from TCGA to identify dysregulated lncRNAs. A novel lncRNA, RP11-439C15.4, was significantly downregulated in HCC tissue compared with normal tissue ( Figure 1A,B) and was positively correlated with patient prognosis ( Figure 1C). To validate these findings, RP11-439C15.4 expression was further examined in 108 HCC tumor tissues and paired non-tumor tissues collected from our clinical center. Consistent with the results obtained from the TCGA, RP11-439C15.4 was indeed significantly downregulated in HCC tissues ( Figure 1D). Patients were divided into RP11-439C15.4-439C15.4-high ( *n*  = 58) and RP11-439C15.4-low ( *n*  = 58) groups on the basis of RP11-439C15.4 expression levels and related clinical information. The correlations between RP11-439C15.4 levels and key clinical indicators and HCC patient prognosis were then analyzed. As shown in Table 1 and Figure 1E,F, patients with lower RP11-439C15.4 expression levels were significantly associated with a more advanced tumor-node-metastasis (TNM) stage ( *P*  < 0.001), a higher rate of tumor invasion ( *P*  = 0.002), and shorter overall survival (OS) and relapse-free survival (RFS) time. Moreover, receiver operating characteristic (ROC) curves generated from the TCGA dataset ( Figure 1G) and data from our clinical center ( Figure 1H) demonstrated that RP11-439C15.4 levels exhibited significant diagnostic value for HCC. Collectively, these results suggest that RP11-439C15.4 is dysregulated in HCC and may be involved in HCC progression.

**Table:** **Table 1** Correlation between RP11-439C15.4 expression levels and the clinicopathologic characteristics of 116 cases of HCC patients

Characteristics	RP11-439C15.4 expression		Pearson’s chi-square test ( *P* value)
Low	High	
Gender				
Male	56	47		0.008
Female	2	11	
Age (y)				
≥ 60	15	26		0.033
< 60	43	32	
AFP				
< 20	17	28		0.036
≥ 20	41	30	
Cirrhosis				
No	27	28		0.852
Yes	31	30	
Differentiation				
Low	11	13		0.647
Moderate/high	47	45	
Tumor size				
< 5cm	24	26		0.708
≥ 5cm	34	32	
Tumor number				
Single	47	52		0.189
Multiple	11	6	
TNM stage				
I	17	36		< 0.001
II	14	8	
III	16	14	
IV	11	0	
Invasion				
No	21	38		0.002
Yes	37	20

### Investigation of the routine characteristics of RP11-439C15.4 in HCC

**Figure 2:**
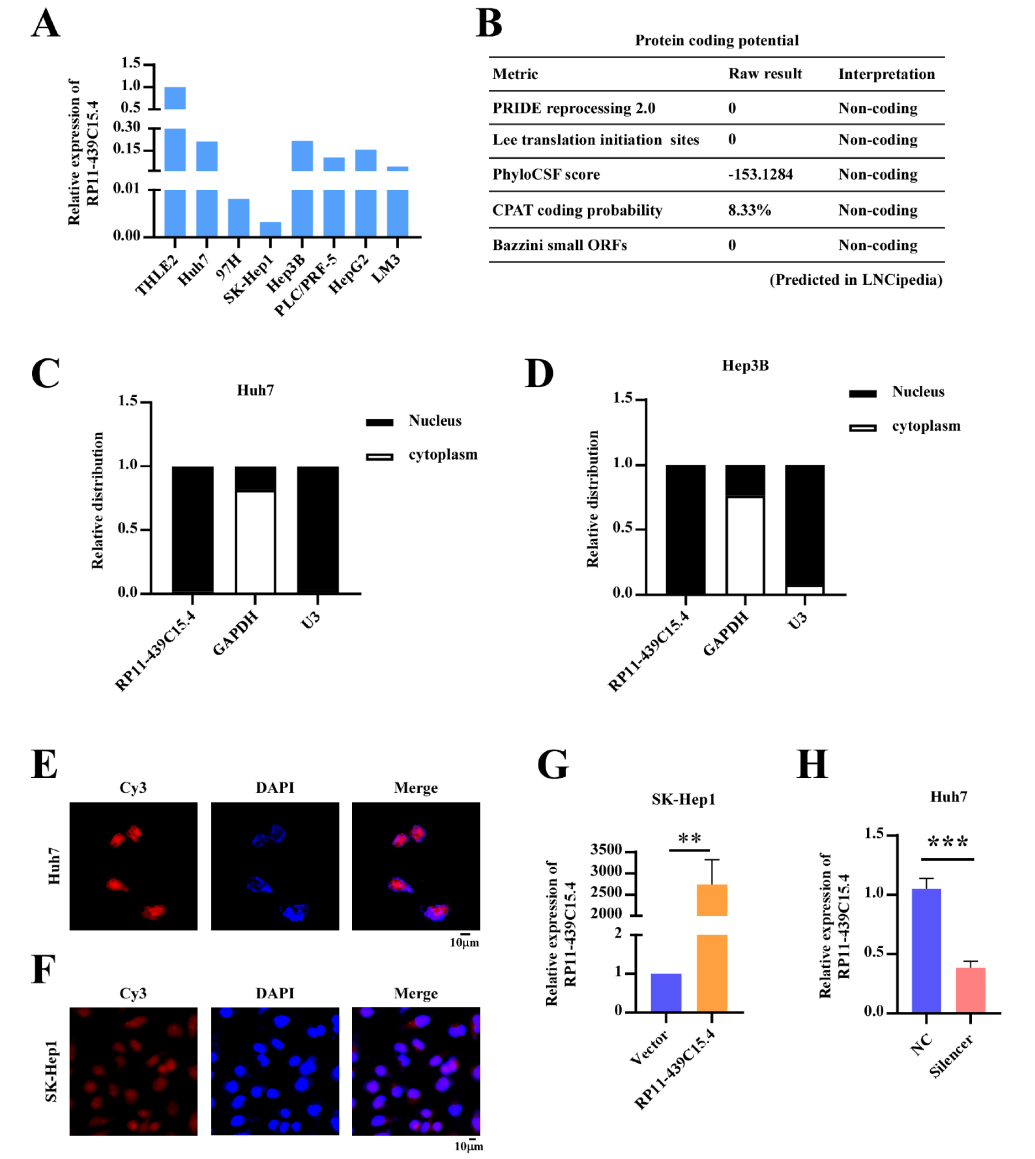
Figure 2 Protein-coding capacity and intracellular localization of RP11-439C15.4 in HCC (A) Analysis of RP11-439C15.4 expression in normal hepatocytes (THLE2) and HCC cell lines (Huh7, 97H, SK-Hep1, Hep3B, PLC/PRF-5, HepG2, LM3) via qRT-PCR. (B) Protein coding potential was analyzed via the LNCipedia platform (https://lncipedia.org). (C,D) The predominant subcellular localization of RP11-439C15.4 was determined through nuclear-cytoplasmic RNA fractionation in Huh7 and Hep3B cells. (E,F) Representative images of FISH assays showing the intracellular localization of RP11-439C15.4 in Huh7 (E) and SK-Hep1 cells (F). Scale bar=10 μm. (G) Stable overexpression of RP11-439C15.4 in SK-Hep1 cells confirmed by qRT-PCR. (H) Successful silencing of RP11-439C15.4 in Huh7 cells confirmed by qRT-PCR assays. P < 0.05 was considered statistically significant. **P < 0.01 and ***P < 0.001.

As shown in Figure 1, low RP11-439C15.4 expression was detected in HCC tissues. In addition to tissue specimens, common HCC cell lines (Huh7, LM3, Hep3B, PLC/PRF-5, HepG2, and SK-Hep1) and normal liver cells (THLE2) were used to assess RP11-439C15.4 expression. qRT-PCR revealed significantly lower RP11-439C15.4 expression in HCC cells than in THLE2 cells ( Figure 2A), which further confirmed that RP11-439C15.4 was downregulated in HCC. These results suggest that RP11-439C15.4 may be involved in the progression of HCC. Prior to formally investigating the function of RP11-439C15.4 in HCC, its routine characteristics (protein coding ability, subcellular localization, *etc*.) were investigated. Analysis via the LNCipedia platform ( https://lncipedia.org/) indicated a lack of protein-coding potential for RP11-439C15.4 on the basis of various algorithms ( Figure 2B). Nuclear and cytoplasmic RNA isolation assays demonstrated predominant nuclear localization of RP11-439C15.4 ( Figure 2C,D). FISH assays further confirmed this nuclear localization ( Figure 2E,F). The qRT‒PCR results ( Figure 2A) revealed that SK-Hep1 cells presented the lowest RP11-439C15.4 expression among the six HCC cell lines, whereas Huh7 cells presented relatively high RP11-439C15.4 expression. In accordance with the conventional strategy of using cell lines to study the function of a specific gene in cancers [ 10, 11] , SK-Hep1 cells were selected for stable RP11-439C15.4 overexpression, and Huh7 cells were used for RP11-439C15.4 knockdown experiments. The success of overexpressing or silencing RP11-439C15.4 in the indicated cells was validated via qRT-PCR assays and is shown in Figure 2G,H.

### RP11-439C15.4 inhibits HCC proliferation, invasion, and migration and enhances HCC sensitivity to sorafenib *in vitro*

Using RP11-439C15.4-overexpressing and -silenced HCC cell models, the effects of RP11-439C15.4 on HCC proliferation were initially assessed via CCK8, colony formation, and EdU incorporation assays. As shown in Figure 3A, [Fig FIG3] C, [Fig FIG3] E; Supplementary Figure S1A and Supplementary Figure S1C, RP11-439C15.4 knockdown induced a significant increase in HCC cell proliferation. Conversely, RP11-439C15.4 overexpression markedly suppressed HCC cell proliferation ( Figure 3B,D,F; Supplementary Figure S1B and Supplementary Figure S1D). Subsequently, transwell and wound healing assays were used to evaluate the influence of RP11-439C15.4 on HCC cell invasion and migration. As shown in Figure 3G,I and Supplementary Figure S1E,G, RP11-439C15.4 silencing significantly promoted HCC cell invasion and migration, whereas RP11-439C15.4 overexpression had the opposite effect ( Figure 3H,J and Supplementary Figure S1F,H). Sorafenib, a multikinase inhibitor, is a first-line treatment for advanced HCC that can effectively induce cell death. However, many patients develop resistance to this drug. Understanding the mechanisms underlying sorafenib resistance and exploring novel therapeutic approaches are crucial for clinical management. This study also investigated whether RP11-439C15.4 affects HCC cell sensitivity to sorafenib. As shown in Figure 3K,L and Supplementary Figure S1I,J, RP11-439C15.4 upregulation significantly enhanced the cytotoxic effects of sorafenib on HCC cells.

**Figure FIG3:**
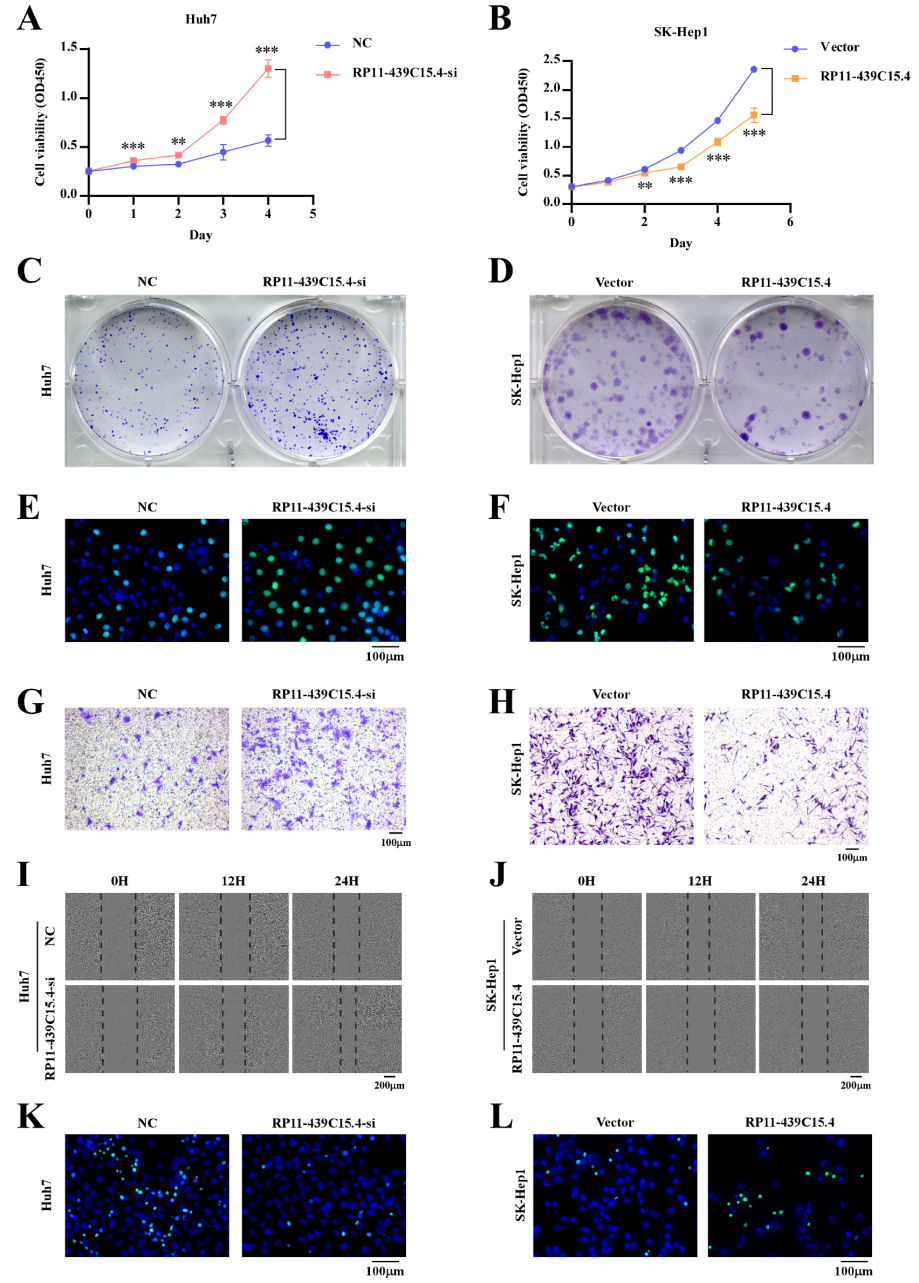
Figure 3 RP11-439C15.4 inhibits the proliferation, invasion and migration of HCC cells and increases the sorafenib sensitivity of HCC cells (A,B) CCK8 assays showing the growth curves of Huh7 and SK-Hep1 cells with different RP11-439C15.4 expression levels. (C,D) Representative images of the colony formation assays of the indicated cells. (E,F) Representative images of EdU-stained cells showing the percentage of EdU-positive cells among the indicated cells. Scale bar = 100 μm. (G,H) Transwell assays were used to assess the invasion abilities of the indicated cells. Scale bar = 100 μm. (I,J) A scratch wound healing assay was used to assess the migration abilities of the indicated cells. Scale bar = 200 μm. (K,L) Representative images of TUNEL staining results showing cell death in the indicated cells treated with sorafenib (10 μM, 24 h). Scale bar = 100 μm. P < 0.05 was considered statistically significant. **P < 0.01 and ***P < 0.001.

### RP11-439C15.4 inhibits HCC tumorigenicity and metastasis *in vivo*

**Figure 4:**
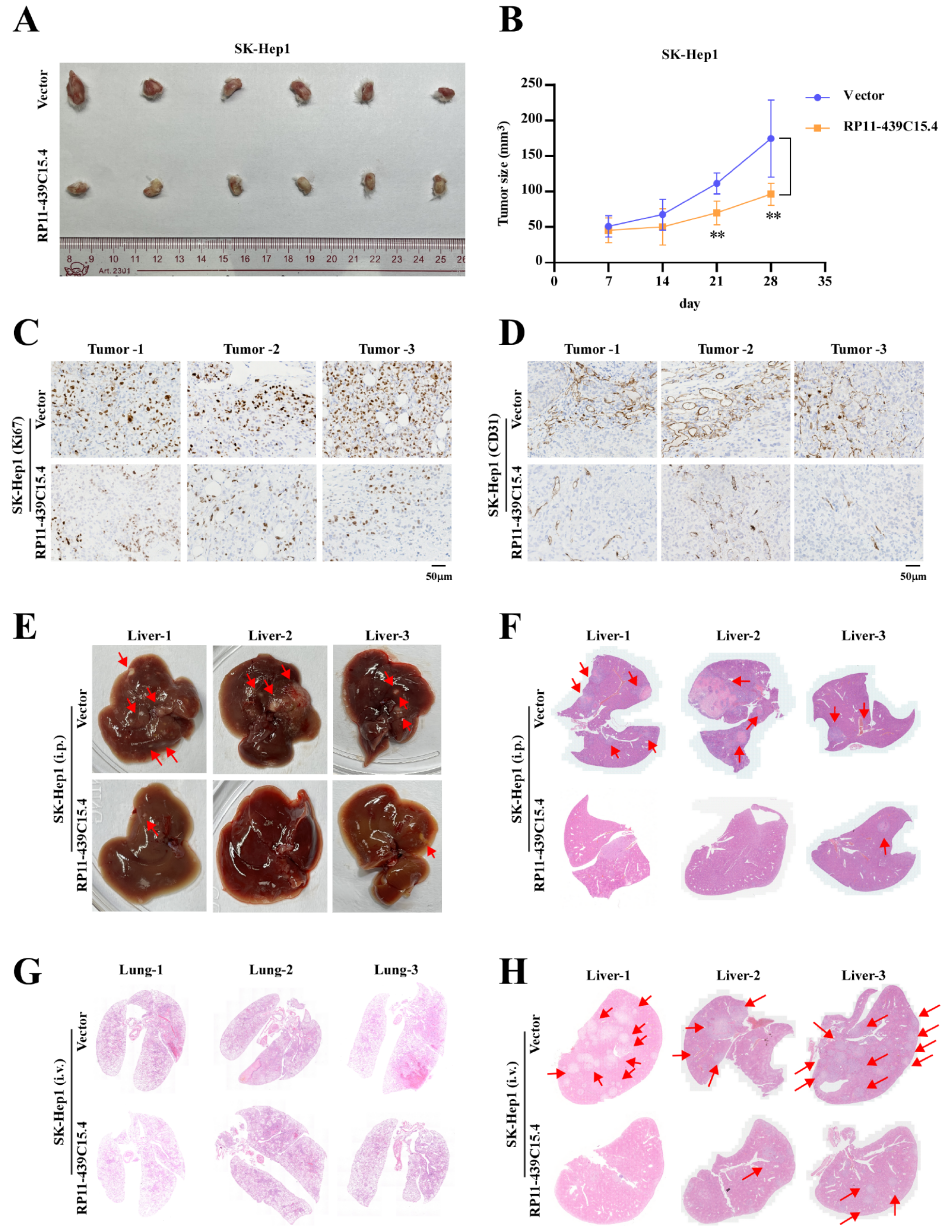
Figure 4 RP11-439C15.4 inhibits tumor growth and metastasis in NCG mice (A,B) Representative images of tumor size and growth curves of the indicated cells. (C,D) Representative images of Ki67 staining and CD31 staining assays showing the influence of RP11-439C154.4 on HCC cell proliferation and angiogenesis. Scale bar = 50 μm. (E) Overview images of liver tissues showing the tumor foci in each group. The red arrow indicates the location of the tumor foci. (F) Representative images of HE staining assays showing the pathological alterations in the livers of each group. The red arrow indicates the location of the tumor foci. (G,H) Representative images of HE staining assays showing the pathological alterations in the lungs and livers of the indicated groups. The red arrow indicates the location of the tumor foci. P < 0.05 was considered statistically significant. **P < 0.01.

On the basis of our *in vitro* results, *in vivo* tumor xenograft and lung metastasis models using SK-Hep1 cells (Vector/RP11-439C15.4) were generated to evaluate the effects of RP11-439C15.4 on HCC tumorigenicity and metastasis. As shown in Figure 4A,B, RP11-439C15.4 overexpression significantly decreased the tumor size and growth rate. Ki67 staining of tumor tissues further validated the effect of RP11-439C15.4 on tumor proliferation. As shown in Figure 4C and Supplementary Figure S2A, the Ki67 staining scale and intensity in tumor tissues from the RP11-439C15.4 high-expression group were significantly lower than those in the control group. Given the importance of angiogenesis in tumor growth and metastasis, the effect of RP11-439C15.4 on HCC angiogenesis was also examined. As shown in Figure 4D, RP11-439C15.4 overexpression significantly inhibited angiogenesis in tumor tissues. Given that SK-Hep1 is a type of HCC cell with strong metastatic ability, we further examined the migration and colonization abilities of the indicated HCC cells in liver tissues. Gross liver examination ( Figure 4E) revealed that RP11-439C15.4 overexpression significantly reduced tumor nodule formation, a finding further confirmed by H&E staining ( Figure 4F). On the basis of these results, we also constructed lung metastasis models with SK-Hep1 cells (Vector/RP11-439C15.4). As shown in Supplementary Figure S2B and Figure 4G, while both SK-Hep1 cell lines (Vector/RP11-439C15.4) formed colonization foci in mouse lungs, the number and area of foci formed by SK-Hep1-RP11-439C15.4 cells were significantly smaller than those formed by control cells. In addition, the formation of liver metastases was significantly reduced by RP11-439C15.4 overexpression ( Supplementary Figure S2C and Figure 4H). Collectively, these *in vivo* results demonstrated that RP11-439C15.4 could significantly inhibit HCC tumorigenicity and metastasis.

### P11-439C15.4 can interact with DHX9 and accelerate its degradation

**Figure 5:**
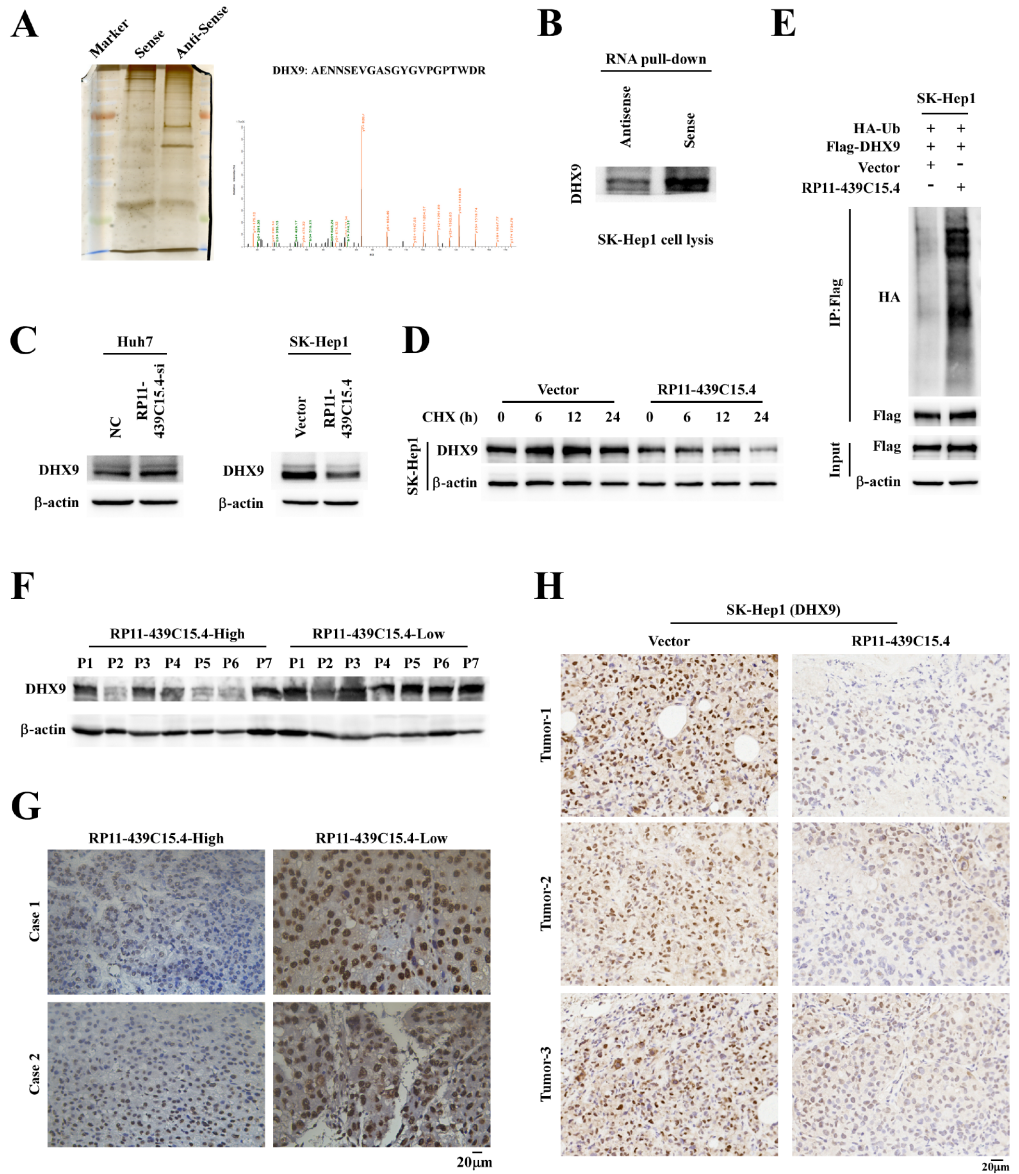
Figure 5 RP11-439C15.4 interacts with DHX9 and enhances DHX9 degradation (A) Identification of the binding partners of RP11-439C15.4 via RNA pull-down assays with LC-MS/MS. The right image shows the mass spectrum of DHX9. (B) RNA pull-down assays further confirmed the interaction of RP11-439C15.4 with DHX9. (C) Western blotting assays showing the effects of silencing/overexpressing RP11-439C15.4 on the protein levels of DHX9. (D) Cycloheximide (CHX) chase assay results showing that RP11-439C15.4 overexpression accelerated DHX9 degradation. (E) Co-IP assays showing the impact of RP11-439C15.4 on DHX9 ubiquitination. (F) Western blotting assays showing the expression of DHX9 in HCC tissues with different RP11-439C15.4 levels. (G) Representative images of DHX9 IHC staining in HCC tissues with different RP11-439C15.4 levels. Scale bar =20 μm. (H) Representative images of DHX9 IHC staining results showing the influence of RP11-439C15.4 on the expression of DHX9 in xenograft tumor tissues. Scale bar = 20 μm.

Previous studies have shown a close relationship between lncRNA function and intracellular localization. Cytoplasmic lncRNAs can function by binding proteins or acting as miRNA sponges, whereas nuclear lncRNAs tend to function through protein binding. As shown in Figure 2, RP11-439C15.4 is located primarily in the nucleus. To elucidate the molecular mechanism underlying RP11-439C15.4 function in HCC, RNA pull-down assays combined with liquid chromatography-tandem mass spectrometry (LC-MS/MS) were performed to identify RP11-439C15.4 binding partners, revealing an interaction with DHX9 ( Figure 5A). This interaction was further validated by RNA pull-down assays in SK-Hep1 cells ( Figure 5B). DHX9, a member of the DEAH-containing family of RNA helicases, catalyzes the ATP-dependent unwinding of double-stranded RNA and DNA-RNA complexes. Previous studies [ 12– 15] have shown that DHX9 is upregulated in HCC and plays important roles in the progression and drug resistance of HCC. We also used HCC samples from our clinical center to confirm that the expression level of DHX9 in HCC tumor tissues was significantly greater than that in adjacent nontumor tissues ( Supplementary Figure S3A,B), which was consistent with previous research. We hypothesized that RP11-439C15.4 functions in HCC through interaction with DHX9. The effects of RP11-439C15.4 on DHX9 protein levels were subsequently assessed. As shown in Figure 5C, RP11-439C15.4 silencing significantly increased DHX9 levels, whereas RP11-439C15.4 overexpression was accompanied by a significant decrease in DHX9 levels. Next, we investigated whether the binding of RP11-439C15.4 and DHX9 affects the stability of DHX9. Half-life experiments revealed that RP11-439C15.4 overexpression significantly shortened the half-life of DHX9 ( Figure 5D). DHX9 degradation is primarily mediated by the ubiquitin-proteasome system. Therefore, the impact of RP11-439C15.4 on DHX9 ubiquitination was evaluated. As depicted in Figure 5E, RP11-439C15.4 overexpression notably increased DHX9 ubiquitination in HCC cells. These findings suggest that RP11-439C15.4 can bind to DHX9 and increase DHX9 ubiquitination in HCC cells, thereby accelerating DHX9 degradation. To further validate the regulatory effect of RP11-439C15.4 on DHX9, we detected DHX9 expression in HCC tissues and xenograft tumor tissues formed from SK-Hep1 cells with different RP11-439C15.4 levels. The results of western blotting and IHC staining assays in HCC tissues revealed that high levels of RP11-439C15.4 were usually accompanied by low expression of DHX9, and vice versa ( Figure 5F,G). IHC staining of xenograft tumor tissues further demonstrated that RP11-439C15.4 overexpression significantly downregulated DHX9 levels in tumor tissues, as evidenced by a decreased DHX9-positive staining ratio ( Figure 5H) and reduced staining intensity ( Supplementary Figure S3C).

### DHX9 is essential for RP11-439C15.4-mediated inhibition of HCC proliferation

**Figure 6:**
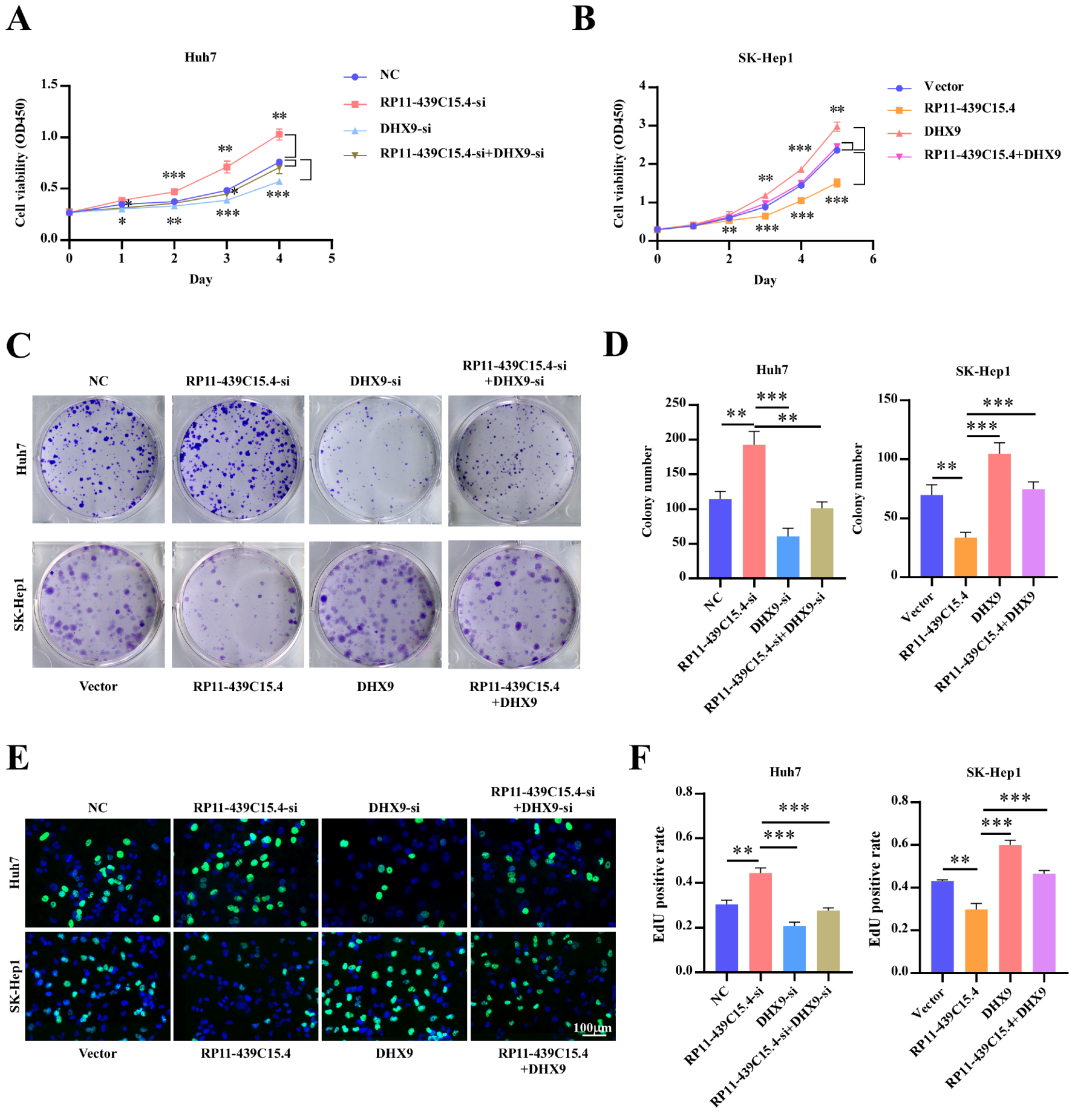
Figure 6 RP11-439C15.4 depends on DHX9 to regulate the proliferation of HCC cells (A,B) CCK8 assays showing the growth curves of Huh7 and SK-Hep1 cells with different RP11-439C15.3 and DHX9 expression levels. (C,D) Representative images (C) and relative quantification (D) of the colony formation ability of Huh7 and SK-Hep1 cells subjected to the indicated treatments. (E,F) Representative images (E) and relative quantification (F) of the results of the EdU incorporation assays of the indicated cells. Scale bar = 100 μm.*P < 0.05, **P < 0.01 and ***P < 0.001.

To test the hypothesis that RP11-439C15.4 functions in HCC by regulating DHX9, DHX9 was overexpressed or silenced in the indicated HCC cells ( Supplementary Figure S4A,B). The effect of altered DHX9 protein levels on RP11-439C15.4-mediated inhibition of HCC cell proliferation was initially examined. CCK8 assays ( Figure 6A,B) revealed that *DHX9* knockdown in RP11-439C15.4-silenced cells significantly reversed the increased proliferation ability of HCC cells induced by RP11-439C15.4 silencing, and vice versa. Colony formation and EdU incorporation assays yielded consistent results ( Figure 6C–F). These results demonstrated that the RP11-439C15.4-mediated inhibition of HCC cell proliferation was dependent on its regulation of DHX9 protein level.

### RP11-439C15.4 is dependent on DHX9 for the regulation of invasion and migration in HCC cells

**Figure 7:**
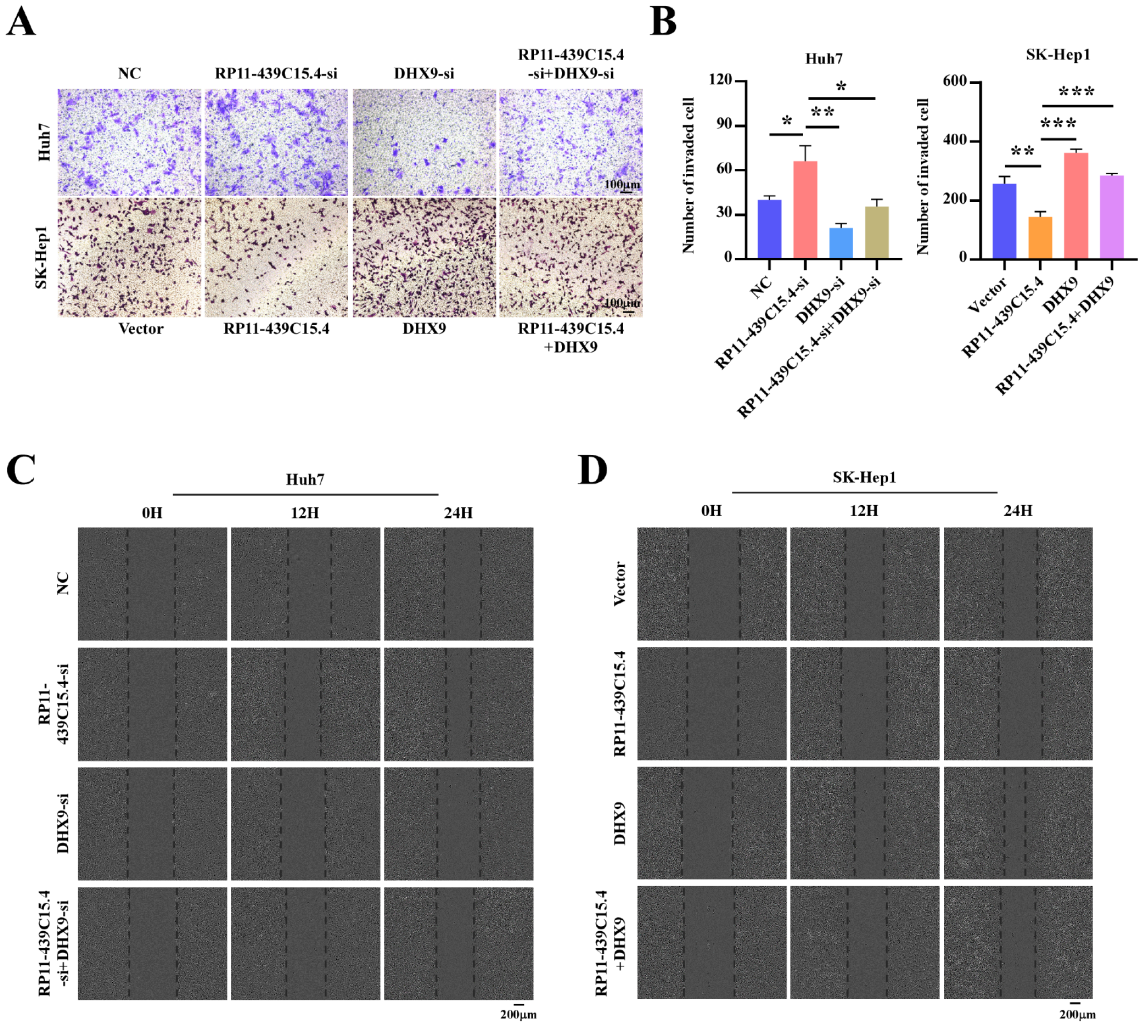
Figure 7 RP11-439C15.4 regulates the invasion and migration abilities of HCC cells in a DHX9-dependent manner (A,B) Representative images (A) and relative quantification (B) for the Transwell assays of the indicated cells. Scale bar = 100 μm. (C-D). Representative images from the wound-healing assays of Huh7 (C) and SK-Hep1 cells (D) with the indicated treatments. Scale bar = 200 μm. *P < 0.05, **P < 0.01 and ***P < 0.001.

To further investigate the role of DHX9 in the RP11-439C15.4-mediated suppression of HCC cell invasion and metastasis, transwell and wound healing assays were performed. As shown in Figure 7A–C and Supplementary Figure S4C, *DHX9* silencing, subsequent to RP11-439C15.4 knockdown in HCC cells, effectively reversed the increased invasion and migration induced by RP11-439C15.4 silencing. Conversely, DHX9 overexpression notably diminished the inhibitory effect of RP11-439C15.4 on HCC cell invasion and migration ( Figure 7A,B,D and Supplementary Figure S4D). Collectively, these results demonstrate that DHX9 is required for the RP11-439C15.4-mediated inhibition of HCC cell invasion and migration.

### DHX9 significantly reverses RP11-439C15.4-mediated sorafenib sensitivity in HCC cells

**Figure 8:**
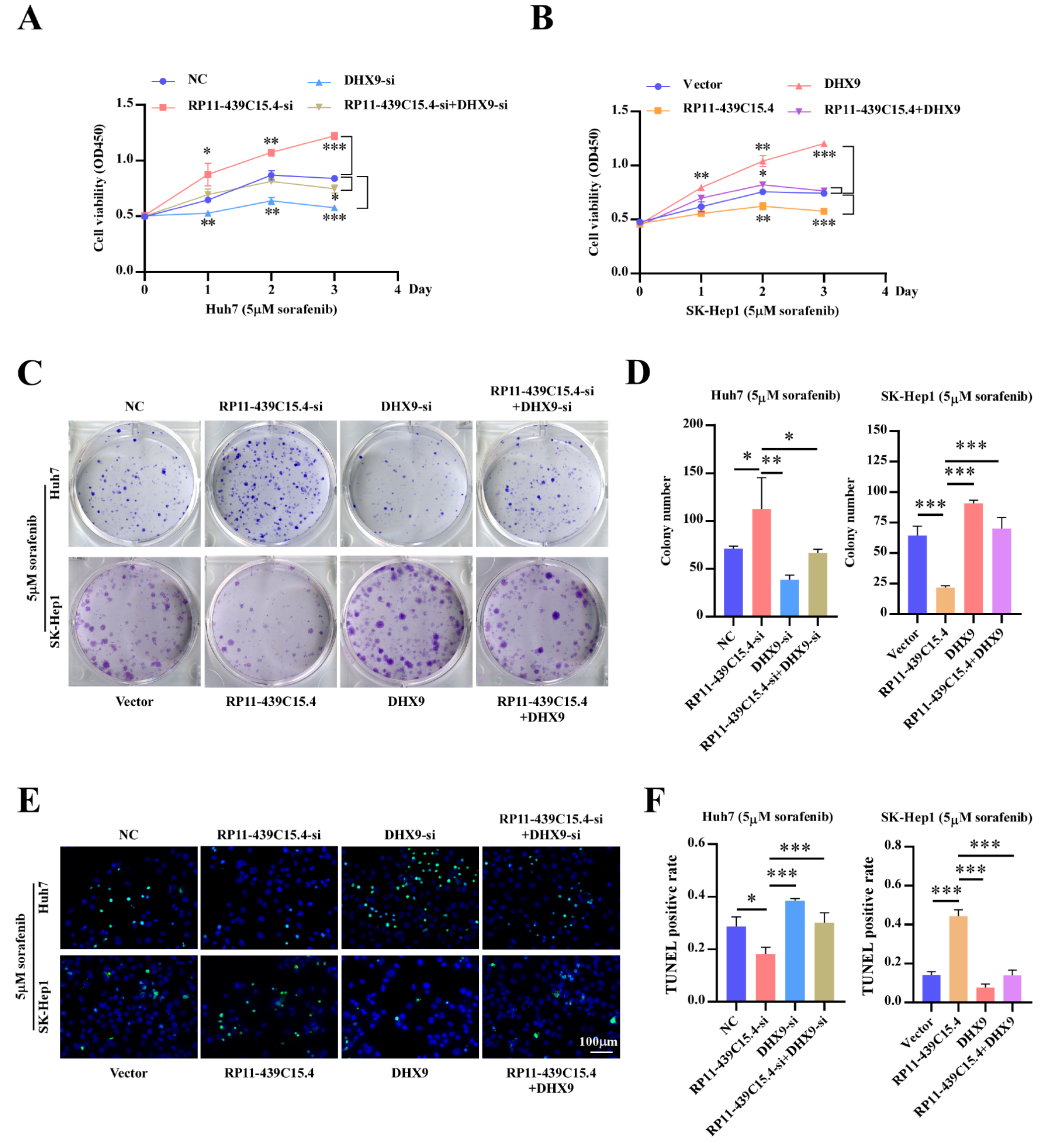
Figure 8 RP11-439C15.4 relies on DHX9 to regulate the sorafenib sensitivity of HCC cells (A,B) CCK8 assays displayed the cell growth curves of Huh7 and SK-Hep1 with indicated treatments. (C,D) Representative images (C) and relative quantification (D) for colony formation assays of HCC cells with indicated treatment. (E,F) Representative images (E) and relative quantification (F) of TUNEL assay results for HCC cells with indicated treatment. Scale bar = 100 μm. *P < 0.05, **P < 0.01 and ***P < 0.001.

Finally, CCK8, colony formation and TUNEL assays were conducted to evaluate the effect of DHX9 on RP11-439C15.4-induced sorafenib sensitivity in HCC cells. The results depicted in Figure 8A,C (upper), D (left), E (upper) and F (left) demonstrated that DHX9 downregulation following RP11-439C15.4 silencing in Huh7 cells effectively reversed the increased sorafenib resistance of HCC cells induced by RP11-439C15.4 knockdown. Conversely, DHX9 upregulation significantly attenuated RP11-439C15.4-induced sorafenib sensitivity, as depicted in Figure 8B,C (lower), D (right), E (lower) and F (right). Taken together, these data indicate that RP11-439C15.4 modulates HCC cell sensitivity to sorafenib in a DHX9-dependent manner.

## Discussion

Increasing evidence indicates that lncRNAs play important roles in the initiation and progression of various cancers, including HCC. For example, Su *et al*. [3] reported that dysregulated lncRNA LINC01089 could promote HCC metastasis by inducing alternative splicing of DIAPH3. Liang *et al*. [15] demonstrated that the lncRNA RP11-295G20.2 could directly bind to PTEN and promote its lysosome-mediated degradation, thereby enhancing HCC progression. Hou *et al*. [16] reported that the lncRNA DIO3OS decreased hepatocellular carcinoma stemness by interfering with NONO-mediated nuclear export of ZEB1 mRNA. Zhang *et al*. [17] reported that the lncRNA NEAT1 upregulated HCC cell sensitivity to ferroptosis by acting as a ceRNA to regulate the miR-362-3p/MIOX axis. Other lncRNAs have also been reported to participate in HCC progression [ 18– 20] . However, owing to the large number of lncRNAs, the functions and corresponding molecular mechanisms of multiple lncRNAs remain unelucidated. In this study, we combined the HCC lncRNA sequencing data obtained from the TCGA and HCC clinical samples to identify a novel lncRNA, RP11-439C15.4, that was significantly downregulated in HCC tumor tissues compared with paired non-tumor tissues. Furthermore, the expression level of RP11-439C15.4 has potential for the diagnosis of HCC. Decreased RP11-439C15.4 expression is associated with a poor prognosis in HCC patients. A series of *in vitro* and *in vivo* experiments revealed that RP11-439C15.4 could significantly inhibit HCC cell proliferation, invasion, and migration and increase cell sensitivity to sorafenib, suggesting a tumor-suppressing role for RP11-439C15.4 in HCC.

LncRNAs can exert their functions in HCC ( *e*. *g*., promoting proliferation, evading apoptosis, accelerating angiogenesis, and conferring drug resistance) through various pathways, including DNA binding and chromatin remodeling, sponging mRNAs and miRNAs, protein interaction and regulation, and even encoding small peptides. In this study, we showed that RP11-439C15.4 could bind with DHX9 and enhance DHX9 ubiquitination, ultimately promoting its degradation. Altering DHX9 levels significantly reversed the effects of RP11-439C15.4 in HCC. Previous studies have shown that lncRNAs can exert functions through various mechanisms, even within the same disease. Zhao *et al*. [21] reported that, in HCC, the nuclear genome-encoded lncRNA MALAT1 interacts with multiple loci on mitochondrial DNA (mtDNA) to influence mitochondrial metabolism by modulating mtDNA CpG methylation and triggering metabolic reprogramming of tumor cells. Hou *et al*. [22] reported that MALAT1 could significantly upregulate VEGF-A expression by sponging miR-140 and that increased VEGF-A, promoted angiogenesis, and facilitated macrophage polarization toward the M2 phenotype. In this study, we investigated the primary roles of RP11-439C15.4 in regulating HCC proliferation, invasion, migration, and sorafenib sensitivity. However, whether RP11-439C15.4 performs other functions ( *e*. *g*., immune escape) and whether it has other downstream targets in addition to DHX9 remain to be further investigated.

DHX9, a helicase enzyme, plays crucial roles in DNA and RNA processing, as well as genome maintenance [ 23, 24] . This versatile enzyme, with multiple domains and functions, can impact cell growth and contribute to tumorigenesis when dysregulated [25]. Previous studies have shown that DHX9 is significantly elevated in HCC tumor tissues compared with normal tissues, enhancing oncogene transcription and facilitating tumor development and metastasis [ 4, 14, 26] . Owing to the complex functions of DHX9 under normal and pathological conditions, directly targeting DHX9 for HCC treatment is not feasible. Elucidating the regulatory mechanisms underlying DHX9 elevation in HCC and targeting specific regulatory molecules may be a viable approach. To date, only Wu *et al*. [27] reported that decreased enzyme activity of the E3 ubiquitin ligase UHRF2 in hepatitis B-associated HCC promotes DHX9 protein stability, thereby increasing DHX9 protein levels. Here, we demonstrated that PR11-439C15.4 can bind with DHX9 and increase the ubiquitination of DHX9, eventually increasing its degradation. The significant decrease in PR11-439C15.4 in HCC disrupts the regulation of DHX9 by PR11-439C15.4, which leads to an increase in DHX9 levels in HCC. This work further elucidates the regulatory network of DHX9 dysregulation in HCC. Thus, targeting PR11-439C15.4 may be used to ameliorate the malignant progression of HCC mediated by DHX9.

In recent years, the COVID-19 pandemic and global health crisis have spurred significant progress in RNA-targeted drug and vaccine design. This progress has propelled RNA research beyond theoretical foundations and toward clinical translation. Given the critical roles of lncRNAs in regulating tumor progression, targeting lncRNAs for tumor treatment and utilizing them for diagnosis and prognosis have garnered increasing attention [ 5, 28– 30] . With the continuous optimization of GalNAc-targeting techniques, the targeted delivery of nucleic acid drugs to the liver has achieved remarkable success [31]. These advancements provide crucial technical support for lncRNA-based disease treatment. In this study, we demonstrated that RP11-439C15.4 is significantly decreased in HCC and that its level has potential for HCC diagnosis and prognosis evaluation. Upregulation of RP11-439C15.4 effectively inhibited HCC malignant progression by inducing DHX9 degradation. The clinical diagnostic and therapeutic value of RP11-439C15.4 warrants further investigation and will be the focus of our future research.

**Figure 9:**
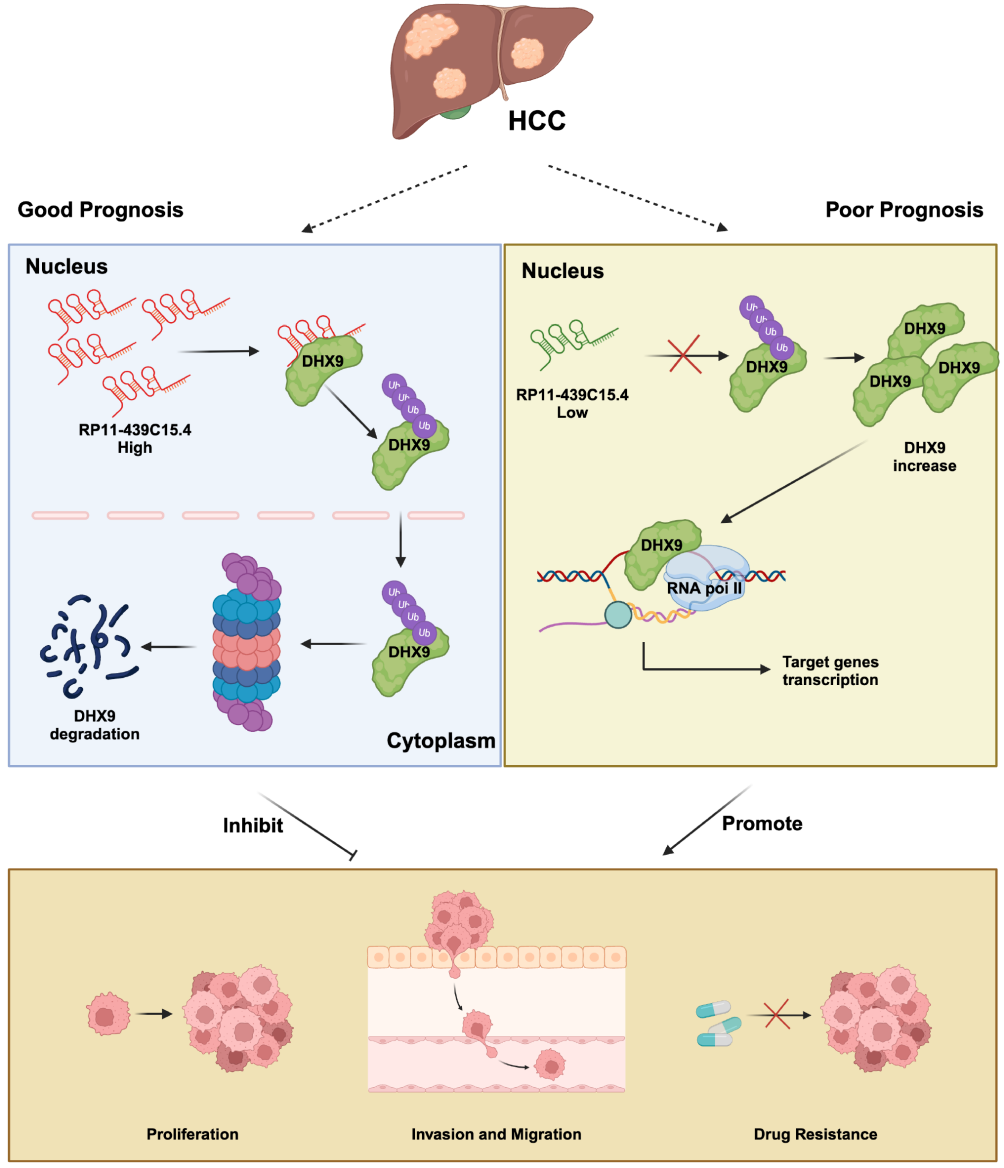
Figure 9 Schematic diagram of a hypothetical model LncRNA RP11-439C15.4 expression positively correlates with HCC patient prognosis. Mechanistically, RP11-439C15.4 interacts with DHX9—an RNA helicase implicated in HCC progression and drug resistance—promoting its ubiquitination and subsequent degradation via the ubiquitin-proteasome pathway. Consequently, elevated RP11-439C15.4 levels suppress HCC cell growth, migration, and invasion while enhancing sorafenib sensitivity, whereas reduced levels accelerate malignant progression.

In conclusion, our work revealed for the first time that PR11-439C15.4 can effectively inhibit HCC proliferation, invasion, migration, and sorafenib resistance by increasing the degradation of DHX9. These findings not only elucidate the functions and related molecular mechanism of PR11-439C15.4 in HCC but also elucidate the regulatory mechanism of DHX9 overexpression in HCC. Moreover, PR11-439C15.4 is a potential therapeutic target and a promising candidate for the evaluation of HCC prognosis ( Figure 9).

## Supporting information

Supplementary_materials

## Supplementary Data

Supplementary data is available at *Acta Biochimica et Biophysica Sinica* online.

## References

[1] Shah M, Sarkar D (2024). HCC-Related lncrnas: roles and mechanisms. Int J Mol Sci.

[2] Llovet J M, Kelley R K, Villanueva A, Singal A G, Pikarsky E, Roayaie S, Lencioni R,
*et al*. Hepatocellular carcinoma.
Nat Rev Dis Primers. 2021, 7: 7. https://doi.org/10.1038/s41572-021-00245-6.

[3] Su T, Zhang N, Wang T, Zeng J, Li W, Han L, Yang M (2023). Super enhancer–regulated LncRNA LINC01089 induces alternative splicing of
*DIAPH3* to drive hepatocellular carcinoma metastasis. Cancer Res.

[4] Liu S, He L, Wu J, Wu X, Xie L, Dai W, Chen L (2021). DHX9 contributes to the malignant phenotypes of colorectal cancer via activating NF-κB signaling pathway. Cell Mol Life Sci.

[5] Huang Z, Zhou JK, Peng Y, He W, Huang C (2020). The role of long noncoding RNAs in hepatocellular carcinoma. Mol Cancer.

[6] Li R, Yan X, Xiao C, Wang T, Li X, Hu Z, Liang J (2024). FTO deficiency in older livers exacerbates ferroptosis during ischaemia/reperfusion injury by upregulating ACSL4 and TFRC. Nat Commun.

[7] Kurien B T, Scofield R H. Western blotting: an introduction.
Methods Mol Biol. 2015, 1312: 17-30. https://doi.org/10.1007/978-1-4939-2694-7_5.

[8] Wisniewski JR, Zougman A, Nagaraj N, Mann M (2009). Universal sample preparation method for proteome analysis. Nat Methods.

[9] Cai J, Li R, Xu X, Zhang L, Lian R, Fang L, Huang Y (2018). CK1α suppresses lung tumour growth by stabilizing PTEN and inducing autophagy. Nat Cell Biol.

[10] Zhang XY, Li SS, Gu YR, Xiao LX, Ma XY, Chen XR, Wang JL (2024). CircPIAS1 promotes hepatocellular carcinoma progression by inhibiting ferroptosis via the miR-455-3p/NUPR1/FTH1 axis. Mol Cancer.

[11] Fang L, Cai J, Chen B, Wu S, Li R, Xu X, Yang Y (2015). Aberrantly expressed miR-582-3p maintains lung cancer stem cell-like traits by activating Wnt/β-catenin signalling. Nat Commun.

[12] Hu Z, Dong L, Li S, Li Z, Qiao Y, Li Y, Ding J (2020). Splicing regulator p54nrb/non–POU domain-containing octamer-binding protein enhances carcinogenesis through oncogenic isoform switch of MYC box-dependent interacting protein 1 in hepatocellular carcinoma. Hepatology.

[13] Chen X, Lin L, Chen G, Yan H, Li Z, Xiao M, He X (2022). High levels of DEAH-Box helicases relate to poor prognosis and reduction of DHX9 improves radiosensitivity of hepatocellular carcinoma. Front Oncol.

[14] Yu J, Xu Q, Wang Z, Yang Y, Zhang L, Ma J, Sun S (2018). Circular RNA cSMARCA5 inhibits growth and metastasis in hepatocellular carcinoma. J Hepatol.

[15] Liang L, Huan L, Wang J, Wu Y, Huang S, He X (2021). LncRNA RP11-295G20.2 regulates hepatocellular carcinoma cell growth and autophagy by targeting PTEN to lysosomal degradation. Cell Discov.

[16] Hou Y R, Diao L T, Hu Y X, Zhang Q Q, Lv G, Tao S, Xu W Y (2023). The conserved LncRNA DIO3OS restricts hepatocellular carcinoma stemness by interfering with NONO-mediated nuclear export of ZEB1 mRNA. Adv Sci.

[17] Zhang Y, Luo M, Cui X, O’Connell D, Yang Y (2022). Long noncoding RNA NEAT1 promotes ferroptosis by modulating the miR-362-3p/MIOX axis as a ceRNA. Cell Death Differ.

[18] Wang F, Hu Y, Wang H, Hu P, Xiong H, Zeng Z, Han S (2023). LncRNA FTO-IT1 promotes glycolysis and progression of hepatocellular carcinoma through modulating FTO-mediated N6-methyladenosine modification on GLUT1 and PKM2. J Exp Clin Cancer Res.

[19] Zhang B, Bao W, Zhang S, Chen B, Zhou X, Zhao J, Shi Z (2022). LncRNA HEPFAL accelerates ferroptosis in hepatocellular carcinoma by regulating SLC7A11 ubiquitination. Cell Death Dis.

[20] Xu K, Xia P, Gongye X, Zhang X, Ma S, Chen Z, Zhang H (2022). A novel lncRNA RP11-386G11.10 reprograms lipid metabolism to promote hepatocellular carcinoma progression. Mol Metab.

[21] Zhao Y, Zhou L, Li H, Sun T, Wen X, Li X, Meng Y (2021). Nuclear-encoded lncRNA MALAT1 epigenetically controls metabolic reprogramming in HCC cells through the mitophagy pathway. Mol Ther Nucleic Acids.

[22] Hou ZH, Xu XW, Fu XY, Zhou LD, Liu SP, Tan DM (2020). Long non-coding RNA MALAT1 promotes angiogenesis and immunosuppressive properties of HCC cells by sponging miR-140. Am J Physiol Cell Physiol.

[23] Ding X, Jia X, Wang C, Xu J, Gao SJ, Lu C (2019). A DHX9-lncRNA-MDM2 interaction regulates cell invasion and angiogenesis of cervical cancer. Cell Death Differ.

[24] Hou P, Meng S, Li M, Lin T, Chu S, Li Z, Zheng J (2021). LINC00460/DHX9/IGF2BP2 complex promotes colorectal cancer proliferation and metastasis by mediating HMGA1 mRNA stability depending on m6A modification. J Exp Clin Cancer Res.

[25] Shi F, Cao S, Zhu Y, Yu Q, Guo W, Zhang S (2021). High expression of DHX9 promotes the growth and metastasis of hepatocellular carcinoma. Clin Lab Anal.

[26] Wang Y, Guo Y, Song Y, Zou W, Zhang J, Yi Q, Xiao Y (2023). A pan-cancer analysis of the expression and molecular mechanism of DHX9 in human cancers. Front Pharmacol.

[27] Wu K, Zhang Y, Liu Y, Li Q, Chen Y, Chen J, Duan C (2023). Phosphorylation of UHRF2 affects malignant phenotypes of HCC and HBV replication by blocking DHX9 ubiquitylation. Cell Death Discov.

[28] Winkle M, El-Daly SM, Fabbri M, Calin GA (2021). Noncoding RNA therapeutics — challenges and potential solutions. Nat Rev Drug Discov.

[29] Liu Y, Liu X, Lin C, Jia X, Zhu H, Song J, Zhang Y (2021). Noncoding RNAs regulate alternative splicing in Cancer. J Exp Clin Cancer Res.

[30] Tan YT, Lin JF, Li T, Li JJ, Xu RH, Ju HQ (2021). LncRNA-mediated posttranslational modifications and reprogramming of energy metabolism in cancer. Cancer Commun.

[31] Kumar V, Turnbull WB (2023). Targeted delivery of oligonucleotides using multivalent protein–carbohydrate interactions. Chem Soc Rev.

