# Supplementary material for: RP11-439C15.4 inhibits the malignant progression of hepatocellular carcinoma via binding to DHX9 and facilitating its degradation: RP11-439C15.4 inhibits HCC progression by downregulating DHX9
Source: Acta Biochim Biophys Sin (Shanghai). 2025 Jul 17;58(2):337–52. doi: 10.3724/abbs.2025122 (PMC12900741; doi:10.3724/abbs.2025122)
Supplement: Supplementary_materials [file Supplementary_materials.docx]

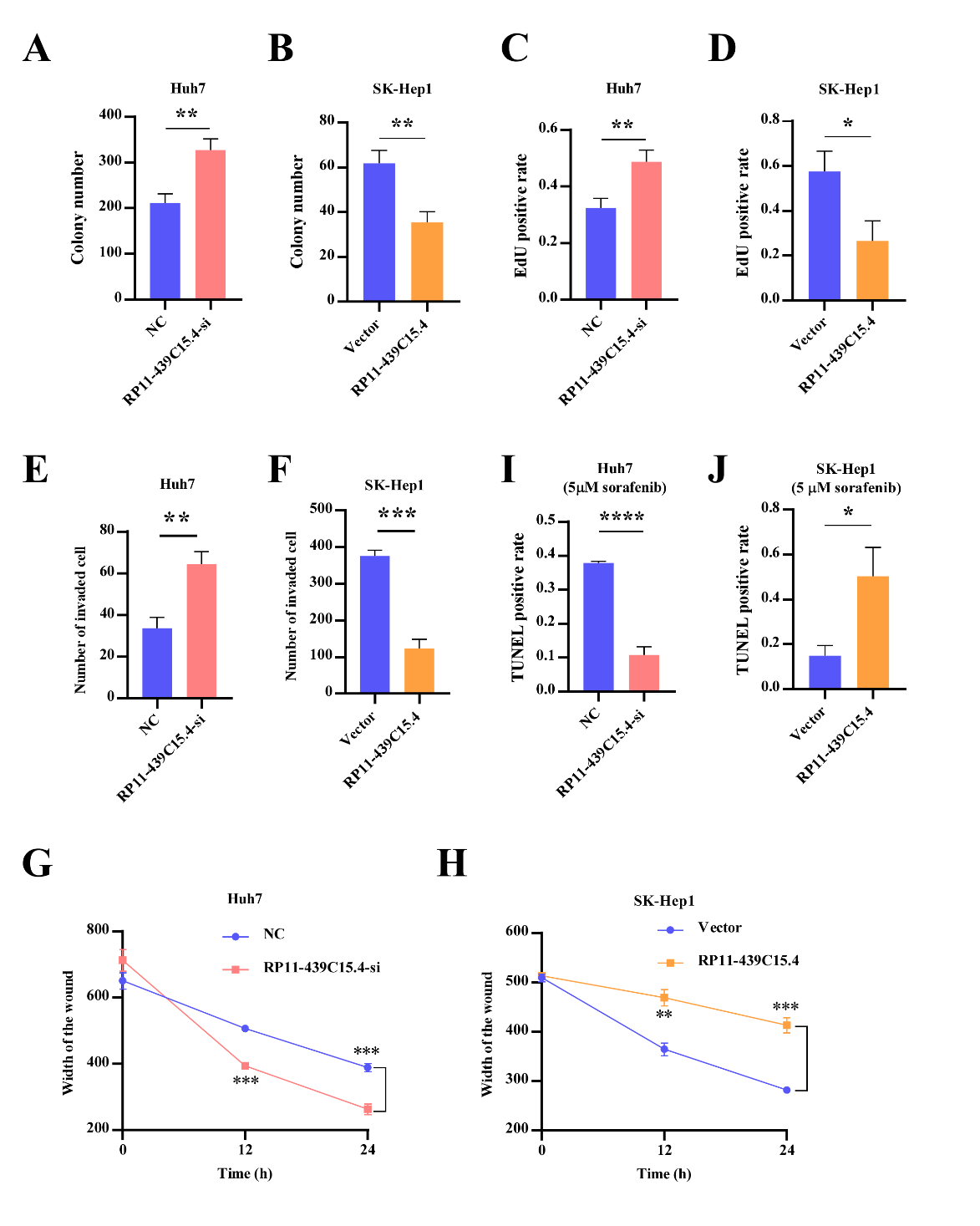


**Supplementary Figure S1. RP11-439C15.4 inhibits HCC cell proliferation, invasion and sorafenib resistance** (A,B) Quantification of the colony formation ability of Huh7 and SK-Hep1 cells with indicated treatments. (C,D) Quantification of the EdU assay results for Huh7 and SK-Hep1 cells subjected to the indicated treatments. (E,F) Quantification of the results of transwell assays of Huh7 and SK-Hep1 cells with indicated treatments. (G,H) Quantification of the wound-healing ability of Huh7 and SK-Hep1 cells with indicated treatments. (I,J) Quantification of TUNEL assays results for Huh7 and SK-Hep1 cells with indicated treatments. *P* < 0.05 was considered statistically significant. **P*< 0.05, ***P*< 0.01, ****P*< 0.001, **** *P*<0.0001.


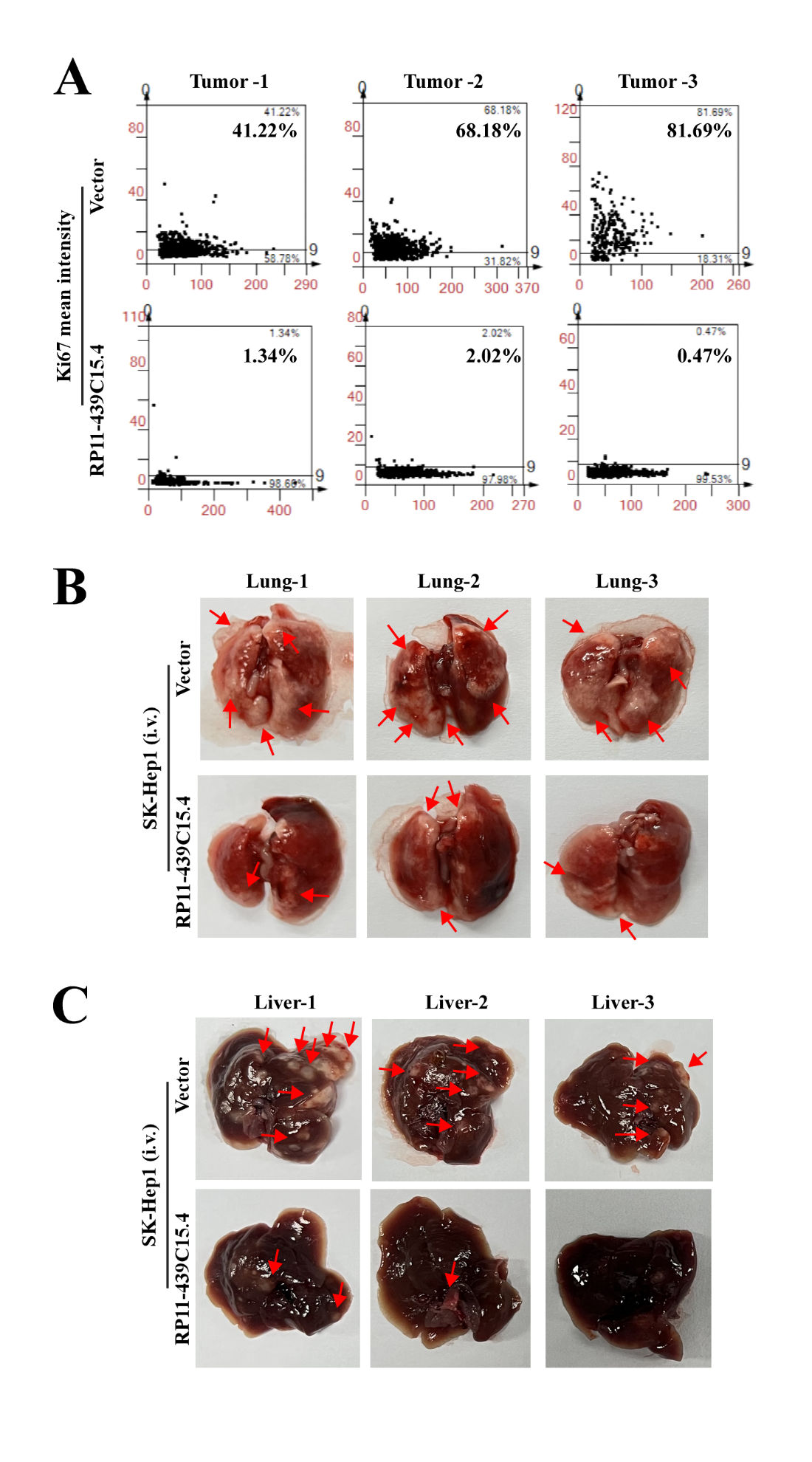


**Supplementary Figure S2. RP11-439C15.4 inhibits HCC tumorigenesis and metastasis** (A) Quantification of Ki67 staining data showing the effect of RP11-439C15.4 on the growth of HCC tumors. (B,C) Overview images of lung (B) and liver (C) tissues showing the tumor foci in each group. The red arrow indicates the location of the tumor foci.


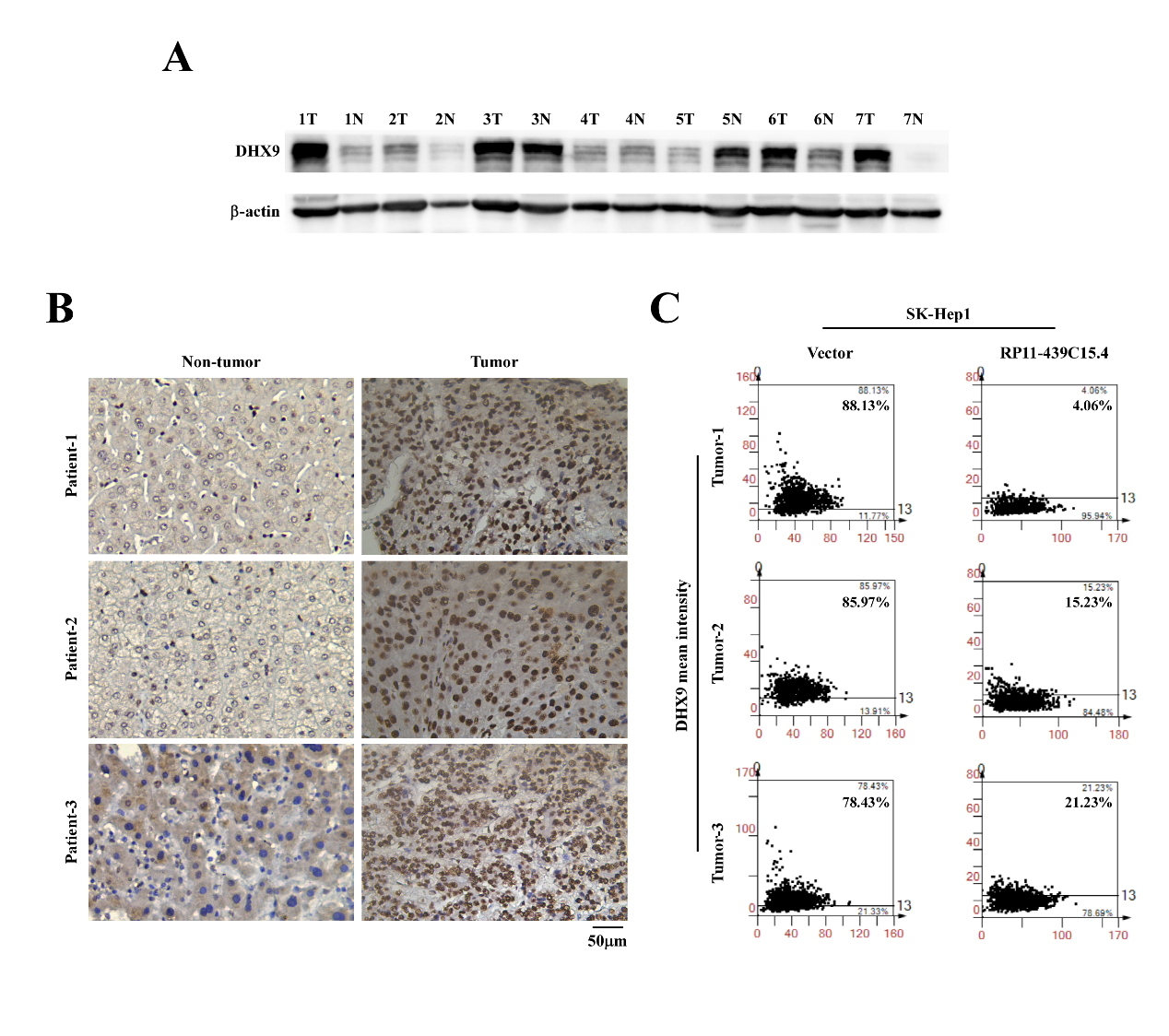


**Supplementary Figure S3. DHX9 is upregulated in HCC, and RP11-439C15.4 influences the expression of DHX9** (A) Western blotting assays showing the expression of DHX9 in HCC tumor tissues and adjacent non-tumor tissues. (B) Representative images of DHX9 IHC staining in HCC tissues and adjacent nontumor tissues. (C) Relative quantification of DHX9 expression via IHC staining in xenograft tumor tissues with different RP11-439C15.4 levels. Scale bar = 50 μm.


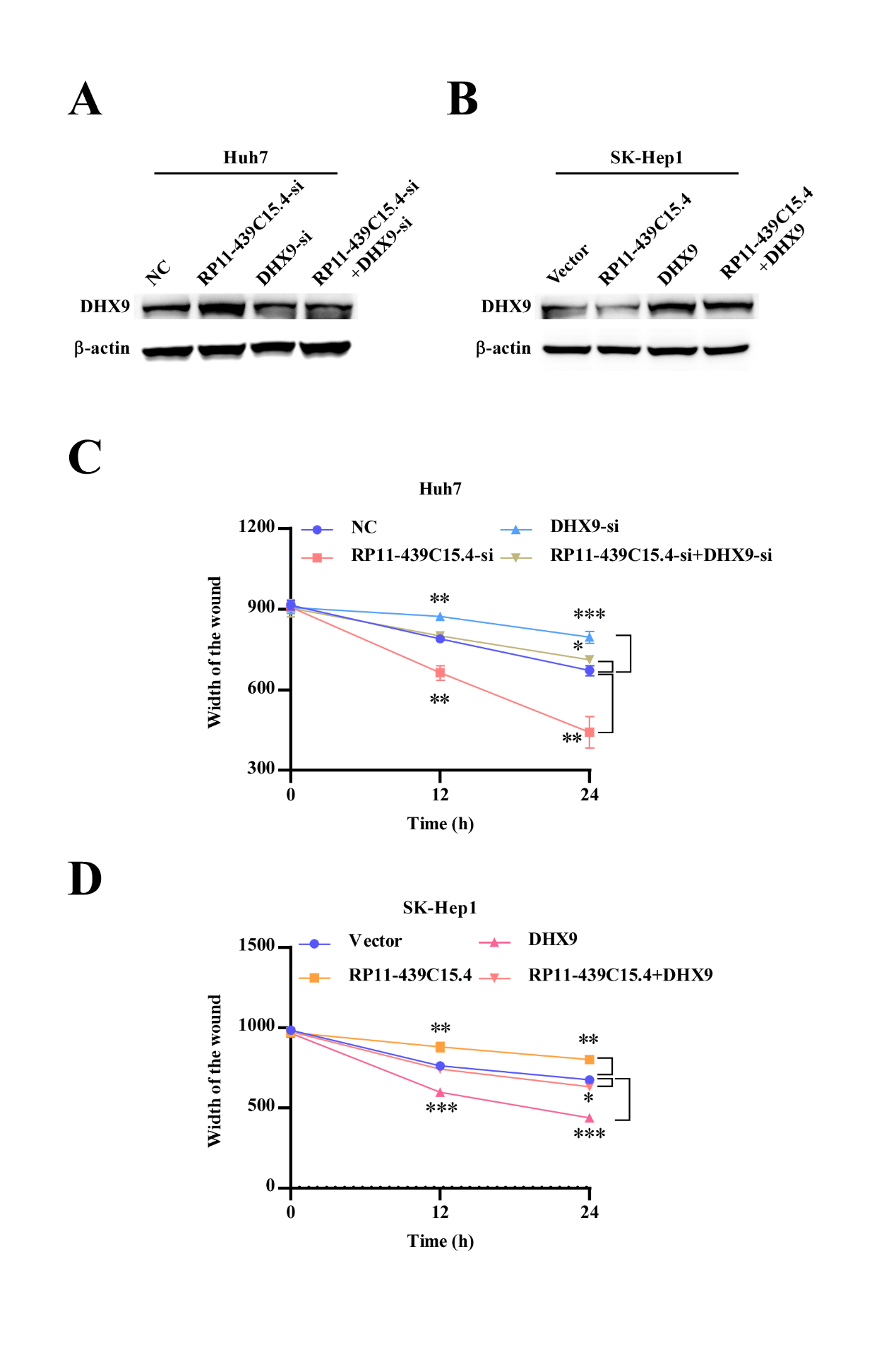


**Supplementary Figure S4.** **Validation of DHX9 silencing or overexpression and the role of DHX9 in RP11-439C15.4-mediated migration inhibition** (A,B) Western blotting assays showing the protein levels of DHX9 in Huh7 (A) and SK-Hep1 cells (B) subjected to the indicated treatments. (C,D) Quantification of the wound-healing ability of Huh7 and SK-Hep1 cells subjected to the indicated treatments. *P*< 0.05 was considered statistically significant. **P*<0.05, ***P*<0.01, ****P*<0.001.

**Supplemental Table S1. List of primers and oligonucleotides used in this study**

| **primer** | | **Sequence (5′-3′)** |
| --- | --- | --- |
| **qRT-PCR primer** | RP11-439C15.4  Forward Primer | TACACATAATGAGCCGTTA |
|  | RP11-439C15.4  Reverse Primer | AGCAGGTACTACTCTTCT |
|  | GAPDH Forward Primer | AAAGGGTCATCATCTCTG |
|  | GAPDH Reverse Primer | GCTGTTGTCATACTTCTC |
|  | U3 Forward Primer | TTCTCTGAGCGTGTAGAGCACCGA |
|  | U3 Reverse Primer | GATCATCAATGGCTGACGGCAGTT |
| **Clone primer** | pLVX(puro)-RP11-439C15.4-up | TCGAGCTCAAGCTTCGAATTCAGGACTCGGGAGCCGCGGCGCGGTG |
|  | pLVX(puro)-RP11-439C15.4-dn | TCGAGCTCAAGCTTCGAATTCGGGTCTGTTTCTGCTATAGTTAACG |
| ***In vitro* Transcription primers** | RP11-439C15.4-T7-sense-up | TAATACGACTCACTATAGGGAGGACTCGGGAGCCGCGGCGCGGTG |
|  | RP11-439C15.4-T7-sense-dn | GGGTCTGTTTCTGCTATAGTTAACG |
|  | RP11-439C15.4-T7-antisense-up | TAATACGACTCACTATAGGGGGGTCTGTTTCTGCTATAGTTAACG |
|  | RP11-439C15.4-T7-antisense-dn | AGGACTCGGGAGCCGCGGCGCGGTG |
| **lnRNA smart silencer** | RiboTM h-RP11-439C15.4  Smart Silencer | TAAAGTTCACAACACGAGAG |
|  |  | AACACGAGAGTGGACGAAAT |
|  |  | TTCTAATTAACGCTGGACCC |
|  |  | CCAGGCTGGGTTCTAATTA |
|  |  | TGCTCATATCCAGAAATGT |
|  |  | ACACGAGAGTGGACGAAAT |
| **DHX9-siRNA**  **NC** | | ACAACAGGAGCTTTATTGC  TTCTCCGAACGTGTCACGT |
